# A Three-Dimensional Engineered Cardiac In Vitro Model: Controlled Alignment of Cardiomyocytes in 3D Microphysiological Systems

**DOI:** 10.3390/cells12040576

**Published:** 2023-02-10

**Authors:** Fatemeh Navaee, Niloofar Khornian, David Longet, Sarah Heub, Stephanie Boder-Pasche, Gilles Weder, Alexander Kleger, Philippe Renaud, Thomas Braschler

**Affiliations:** 1 Microsystems Laboratory-LMIS4, École Polytechnique Fédérale de Lausanne (EPFL), 1015 Lausanne, Switzerland; 2 Faculty of Medicine, Department of Pathology and Immunology, Centre Médical Universitaire (CMU), 1206 Geneva, Switzerland; 3 Institute of Molecular Oncology and Stem Cell Biology, Ulm University Hospital, 89081 Ulm, Germany; 4 Swiss Center for Electronics and Microtechnology (CSEM), 2002 Neuchatel, Switzerland; 5 Interdisciplinary Pancreatology, Department of Internal Medicine 1, Ulm University Hospital, 89081 Ulm, Germany; 6 Organoid Core Facility, Medical Faculty, Ulm University Hospital, 89081 Ulm, Germany

**Keywords:** 3D cell culture, in vitro cardiac model, cardiac cell alignment, 3D hydrogel, decellularized extracellular matrix (ECM), microfabricated grooves

## Abstract

Cardiomyocyte alignment in myocardium tissue plays a significant role in the physiological, electrical, and mechanical functions of the myocardium. It remains, however, difficult to align cardiac cells in a 3D in vitro heart model. This paper proposes a simple method to align cells using microfabricated Polydimethylsiloxane (PDMS) grooves with large dimensions (of up to 350 µm in width), similar to the dimensions of trabeculae carneae, the smallest functional unit of the myocardium. Two cell groups were used in this work; first, H9c2 cells in combination with Nor10 cells for proof of concept, and second, neonatal cardiac cells to investigate the functionality of the 3D model. This model compared the patterned and nonpatterned 3D constructs, as well as the 2D cell cultures, with and without patterns. In addition to alignment, we assessed the functionality of our proposed 3D model by comparing beating rates between aligned and non-aligned structures. In order to assess the practicality of the model, the 3D aligned structures should be demonstrated to be detachable and alignable. This evaluation is crucial to the use of this 3D functional model in future studies related to drug screening, building blocks for tissue engineering, and as a heart-on-chip by integrating microfluidics.

## 1. Introduction

Studies of in vitro cardiac models are critical to improving scientists’ understanding of cardiovascular physiology, diseases, and treatments [1]. They have also provided vital information regarding the recapitulation of the complex heart microenvironment. Now, by studying, comparing, and testing existing and new drug screening strategies on in vitro cardiac models, there is a promising potential to develop novel drugs capable of targeting cardiovascular disease (CVD) which deliver a more precise pharmacological response [2].

A variety of physiological functions are affected by the spatial arrangement of cardiomyocytes and fibroblasts in the extracellular matrix (ECM) in native myocardial tissue, particularly in the generation of contractile force along with biological, electrical, and mechanical functions [3]. The appropriate distribution of cells within this tissue architecture, as well as its three-dimensional (3D) nature, is directly related to operative and active forces [4]. Additionally, the conduction of electrical currents in the heart is anisotropic, as it spreads more rapidly along the long axes of aligned cardiac fibers [5]. It is, therefore, essential to have a precise and realistic understanding of the myocardial fiber architecture and its intimate physiology in order to accurately interpret the electrical stimulus and the mechanics of the heart [6,7,8]. However, the design and building of a 3D in vitro heart model remains a challenge. Therefore, understanding the smallest physiologically relevant and functional myocardium tissue unit in the native heart must be considered as the starting point for aligned cardiac model fabrication. Trabeculae carneae are considered to be the smallest functional units with the same cell composition and mechanical properties as the native heart (Figure S1). They consist of irregular muscular structures that pattern the inner surface of both ventricles of the heart [9] and similar cellular strands fuse during development to constitute the solid myocardium [10]. These structures represent the smallest collections of aligned cardiomyocytes in the heart, with dimensions of 0.5–3 mm in length and 50–500 µm in diameter [11]. Due to its dimensions, trabeculae carneae permits swift diffusion of oxygen and nutrients within these structures [12]. Correspondingly, the vascularization in the trabeculated myocardium is poorer than of the compact myocardium, which is why it is often favored by researchers for the ex vivo studies of ionic, mechanical, and metabolic activity of the cardiac muscle [11,13]. Compared with the 2D cultures, an accessible in-vitro cardiac model which mimics the 3D structure of trabeculae carneae could provide a more realistic, reliable and robust platform for drug screening, and, furthermore, facilitate detailed investigation of physiology, biomechanics, and function of heart. The construction of an in-vitro cardiac model still faces several difficulties after vascularization is considered; among others, cell alignment in three dimensions, cellular mechanical and functional environments, cell connectivity, extracellular matrix and soluble differentiation cues, and local cell composition in terms of stroma and functional cells [3].

Among these challenges, controlling the patterns and mechanical anisotropy of in vitro cell cultures to develop functional tissue is considered primordial [14]. The physical environment indeed affects the function of the cardiomyocyte cells through mechano-transduction processes. Physical cues around the cells are integrated by being converted to intracellular biochemical signals that translate changes in cell function [15]. When an external force is applied to a cell’s ECM, every feature of the ECM is altered and rearranged [15]. Cell alignment, therefore, feeds back into local biomechanics, cellular mechanical function, electrical coupling, and, ultimately, maintenance of a differentiated phenotype. Indeed, in 2D cultures, aligned cardiomyocyte cells were reported to exhibit a substantial expression of genes that impact on cardiomyocyte function and differentiation [16,17,18,19,20,21]. During the development of our model, we primarily focused on implementing a facile cell alignment in 3D that allows the generation of robust, free-standing structures featuring accurate cellular functionality and maintenance of a differentiated phenotype.

Several research groups have investigated the use of micropatterned geometries to induce cell alignment in 3D. Photolithographic hydrogel in situ patterning [3,22] and hydrogel molding in microgrooves [1,23,24] are typically employed in order to demonstrate a general propensity of various cell types, including cardiomyocytes, to align with microfabricated geometrical features. Substrate anisotropy is important as well, since bottom-up 3D fabrication of flexible, anisotropic honeycomb showed improved cardiomyocyte alignment when compared with isotropic control scaffolds [25].

Jointly, these microscale studies indicate that local microgeometry is a critical determinant for 3D cell alignment. The cellular alignment in the native cardiac muscle, however, extends far beyond 10–100 micrometers, which represents the typical size for these microscale experiments. In the framework of so-called Engineered Heart Tissues (EHT), cellular alignment at a macroscopic 1–10 mm scale was studied in large ring-shaped self-condensing structures made from a collagen-Matrigel mix seeded with neonatal rat cardiomyocytes. Strong alignment of the cells along the tangential direction was found throughout the macroscopic scale of the system [26,27]. Despite its efficient traits, the underlying mechanism of alignment from these experiments is poorly understood. Therefore, hydrogel remodeling and ensuing anisotropy during self-condensation, strong metabolic gradients, static and dynamic stress produced by the contractile cells, as well as geometric constraints similar to the microscale experiments should be regarded.

Our study aims to address the challenge of 3D alignment of cells in constructs that accurately replicate the dimensions of trabeculae carneae [28], and to delimit the scale accessible through propagation of alignment from surface topography. We previously developed a composite hydrogel to match the mechanical properties of heart muscle in absence of potentially cytotoxic crosslinkers [29]. We present here a rather simple method to control cell alignment in 3D cell-laden hydrogels that are patterned using microfabricated PDMS grooves with large dimensions (up to 350 µm in width), similar to the dimensions of trabeculae carneae. For this purpose, we molded the composite hydrogel featuring a decellularized cardiac extracellular matrix and fibrin mixed with live cells on the grooved PDMS. Subsequently, we compared this model to non-patterned 3D constructs as well as 2D cell cultures, with and without patterns. Here, two different cell models (either a co-culture of H9c2 and fibroblasts or neonatal cardiac cells) were involved. Beyond alignment, we assessed the functionality of our proposed 3D model by comparing beating rates of the aligned and non-aligned structures. Finally, to evaluate the practicality of the model, the detachability and alignment stability of the 3D aligned structures were demonstrated. These evaluations are critical for use of our 3D functional model in future investigations relating to drug screening, building blocks for tissue engineering, or by applying microfluidic systems in heart-on-chip designs.

## 2. Materials and Methods

### 2.1. Microfabricated Grooves for Cell Alignment

**Figure 1:**
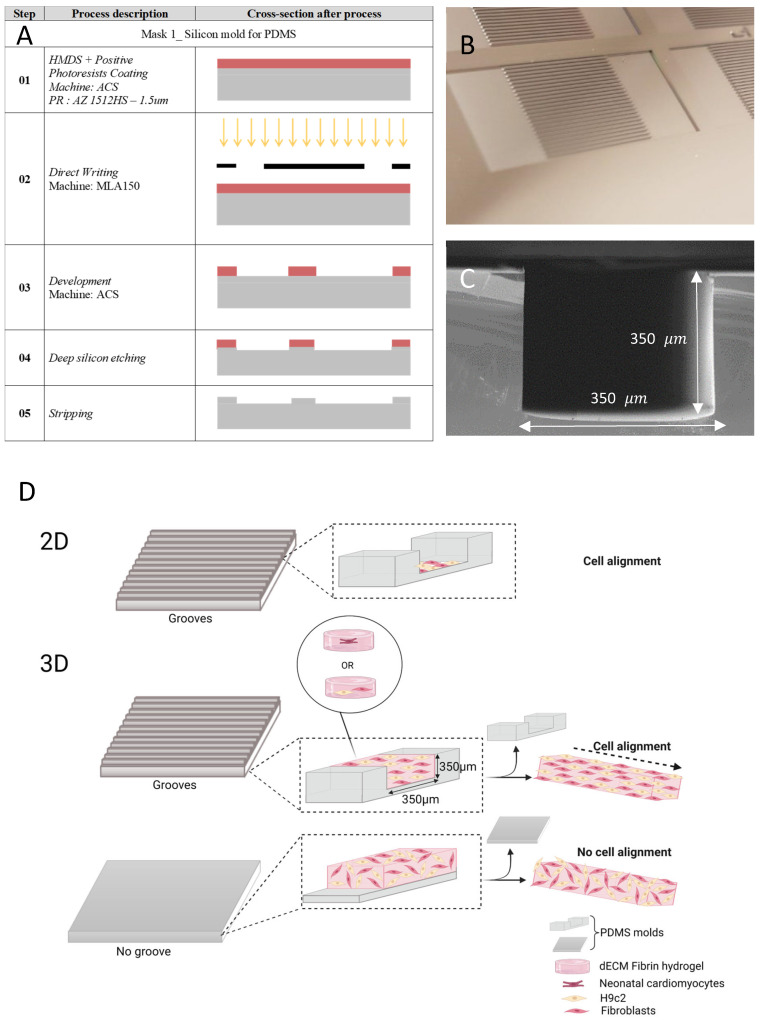
The process of chip fabrication and schematic of the work. (**A**) the main steps of groove microfabrication. (**B**) Etched silicon wafer for fabricating the microgrooves. (**C**) SEM pictures of microfabricated grooves patterned in silicon wafer. (**D**) schematic describing the alignment in 3D cardiac model.

A specific mold was used to generate specific patterns in the hydrogel structure. The preparation of this mold included the design and fabrication of various silicon (Si) wafers, each with uniform microfabricated grooves of different dimensions. Figure 1A illustrates the main steps of the silicon wafer etching. Direct laser photolithography followed by deep silicon etching with the standard Bosch process was employed at the Center of Micronanotechnology (CMi) at Ecole Polytechnique Fédérale de Lausanne (EPFL). Six squared channels featuring different sizes were produced/manufactured (channel size × spacing: 100 μm × 100 μm, 150 μm × 150 μm, 200 μm × 200 μm, 250 μm × 250 μm, 300 μm × 300 μm, and 350 μm × 350 μm, with 1 cm length) on each single-sided 100 mm Si wafer. After wafer fabrication, salinization was conducted/performed by exposing the wafer to trimethylsilyl chloride (TMCS) vapors under a chemical hood. Scanning electron microscopy (SEM) was employed to image the grooves fabricated according to predefined dimensions (Figure 1C). These structured wafers were used to produce the patterned PDMS substrate. Firstly, Sylgard 184 silicone base and Sylgard curing agent (Dow Corning, Horgen, Switzerland) were prepared with the ratio of 10:1, mixed for 1 min at 2000 rpm, degassed for 2 min at 2200 rpm, and then poured onto the silicon wafer, followed by an additional degassing step. PDMS curing was undertaken at 80 °C for two hours, after which the wafer was removed. Afterwards, the PDMS was exposed to oxygen plasma with 100 W power for 60 s to render the surface hydrophilic, before use. Microfabricated grooves successfully employed in accurate determination of cell alignment.

### 2.2. Hydrogel Preparation

A dECM-fibrin hydrogel developed in our laboratory [29] was used as the 3D structure for the cells. The hydrogel, which provides a suitable mechanical and biological environment, was obtained by mixing decellularized extracellular matrix (dECM) and fibrinogen (Sigma, F3879, Buchs, Switzerland) at a final concentration of 5 mg/mL and 26.4 mg/mL, respectively. Gelation occurred by adding Thrombin (Sigma, T1063, 250 U/mL, Buchs, Switzerland) and calcium chloride to the pre-gel solution. The mechanical properties of the hydrogel reflected the range of native cardiac tissue (approx. 20 kPa). Furthermore, the hydrogel can be deployed in investigations of several biological functions such as differentiation, beating rate, and synchronicity in vitro.

### 2.3. Cell Culture

Two cell models were employed. Firstly, the co-culture of H9c2 cells (a cell model used as an alternative for cardiomyocytes) and NOR-10 (fibroblast) cells were used to demonstrate the principle of cell alignment in 3D structures. Secondly, neonatal cardiac cells were involved to investigate the behavior of the cardiomyocytes in terms of their beating and synchronicity.

H9c2 cells, obtained from the European Collection of Authenticated Cell Cultures (ECACC) (Lot# 17A028), were cultivated in Dulbecco’s Modified Eagle Medium (DMEM) (Thermofisher, cat# 41965) supplemented with 10% fetal bovine serum and 1% penicillin and streptomycin in 75 cm^2^ tissue culture flasks at 37 °C and 5% CO_2_ in humidified atmosphere. Cells were passaged before reaching 70–80% confluency to avoid loss of differentiation potential [30]. NOR-10 (ECACC 90112701) cells were obtained from the European Collection of Authenticated Cell Cultures. The cells were cultured in DMEM medium supplemented with 10% fetal bovine serum and 1% penicillin and streptomycin in 75 cm^2^ tissue culture flasks at 37 °C and 5% CO_2_ in an incubator. Splitting occurred before cells reached 70–80% confluency according to the supplier’s notice [31]. For our first cell model, we used co-cultures with a seeding ratio of 30% H9c2 and 70% Nor-10 fibroblasts, inspired by the native fraction of cardiomyocytes in the heart. The second cell model relied on primary rat neonatal cardiomyocytes, which were isolated from Winstar neonatal (P1) rat hearts using the Pierce Primary Cardiomyocyte Isolation Kit, according to the manufacturer’s instructions [32]. Organ harvesting was performed on sacrificed control animals from unrelated experiments, according to license VD 3290.

### 2.4. Cell Seeding on PDMS (2D) and in 3D Hydrogel

**Figure 2:**
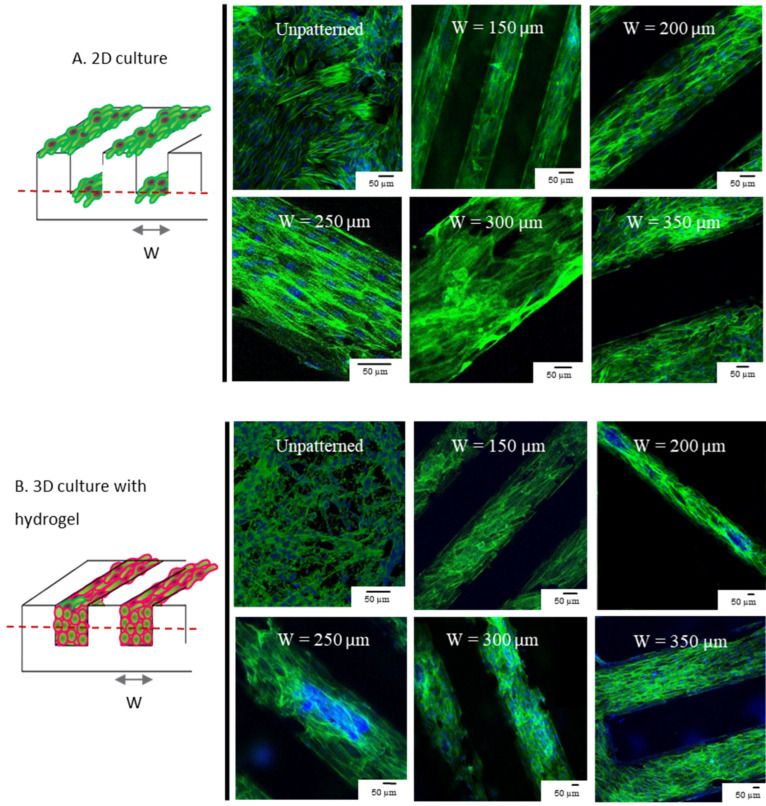
Confocal imaging of cell alignment using microfabricated grooves. (**A**) cells cultured on patterned PDMS molds without hydrogel, creating the 2D stripes. (**B**) cells encapsulated in the dECM-fibrin hydrogel and cultured in patterned PDMS molds in 3D. Cell alignment is observed in all cases, regardless of the structure dimensions, in both 2D and 3D. The red dashed line indicates the confocal image stack. Green: Phalloidin, Blue: DAPI.

To demonstrate the advantage of 3D vs. 2D cultures on cell alignment and functionality, cells were cultured on the grooved PDMS substrate with hydrogel (3D) and without hydrogel (2D) (Figure 2). For the 2D culture, the cell suspension in medium was directly poured/cast onto the plasma-treated microstructured PDMS substrate with initial cell density of 2.5 × 10^5^ cells per cm^2^, followed by direct cell attachment and spreading. For the 3D culture with hydrogel, cells were first mixed with pre-gel with an initial density of 10^6^ cells per mL, followed by the addition of thrombin and calcium chloride to induce gelation. 200 µL cell suspension in pre-gel was then immediately poured onto the PDMS substrate until solidified and patterned hydrogel was achieved. Given the rapid gelling time (<2 min), the cells remained suspended in the viscous gel until solidification. The flat PDMS substrates were used as controls for both 2D and 3D conditions. The experiment was applied to both cell models (the co-culture of H9c2 cells with fibroblasts, and neonatal cardiac cells). Samples were incubated at 37 °C, 5% CO_2_, and medium were changed every two days. The samples were analyzed after 7 days in culture.

### 2.5. Staining and 3D Imaging

Prior to staining, the H9c2 and NOR-10 cell samples were fixed with 4% paraformaldehyde (PFA) for 20 min at room temperature. Then, 0.1% TritonX-100 was added for 30 min at room temperature to permeabilize the cells. Actin filaments were stained by incubating the cells with phalloidin-Atto 488 (1:50) for 45 min at 4 °C. Finally, the phalloidin was removed, washed, and replaced with DAPI (1:2000) for 5 min, for labeling the nuclei. The DAPI was washed with phosphate buffered saline (PBS) (Gibco 2062235) before imaging the cells under a ZEISS LSM 700 inverted confocal microscope following the pattern indicated in Figure 2.

### 2.6. Cell Orientation

**Figure 3:**
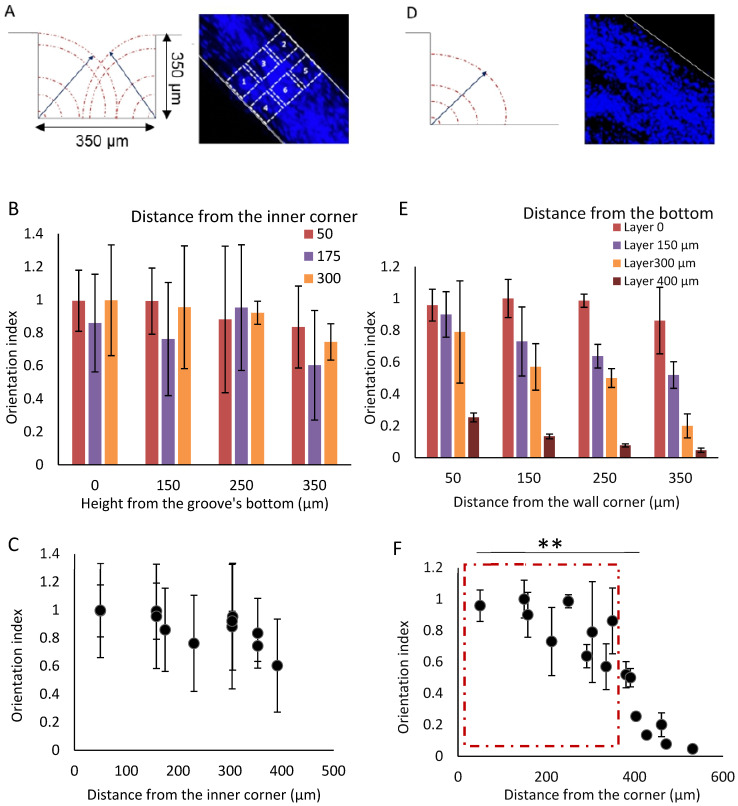
The calculation of the distance of alignment. (**A**) the contact guidance of the corners to improve the cell alignment in a groove. The region of interest (ROI) in a stack of confocal image for measuring the alignment in the cells. Measurements are repeated for several heights. ROI area 100 × 100 µm. Positions: 1 and 2 = near the wall, 3 = center of the groove, 4, 5 and 6 = repeat of analysis in proximal position in the groove. (**B**) the measurement of cell alignment in relation to the distance from the groove’s corner in a groove with 350 µm by 350 µm dimensions. The cell alignment in different height from the bottom of the groove. Two-way ANOVA shows no significant difference in the alignment. (**C**) the orientation index in different distance from the inner corner of the groove. Maximum distance from the corner in this case is 391 µm. (**D**) the contact guidance of the corner in a groove with infinite width. The middle confocal stack of H9c2 cells in co-culture with fibroblasts in 3D dECM-fibrin hydrogel, DAPI staining. The cells are restricted on one side. The height of the groove is 350 µm and the width is 2 mm which can be considered as infinite. (**E**) the measurement of cell alignment in relation to the distance from the groove’s corner in a single edge configuration. The cell alignment in different height from the bottom of the groove. Two-way ANOVA analysis shows significant impact on distance from bottom and wall corner in the groove with infinite width. (**F**) the orientation index at different distances from the inner corner of the groove. Maximum distance with the orientation of the cells is around 250–300 µm from the inner corner. Scale bar: 200 µm. ** = *p* ≤ 0.01.

The quantitative analysis of H9c2 and NOR-10 cell alignment was achieved on a selection of different stacks of confocal images with various heights of up to 350 μm from the bottom of the groove. The maximum distance of the cell alignment in the 3D hydrogel was calculated, and the impact of inner corner contact on the alignment was also assessed using OrientationJ plugin in ImageJ software; three regions of interest (ROI) in each stack with an area of 100 µm × 100 µm close to each wall and in the middle of the stack were identified (Figure 3A). Each experiment was repeated three times. The analysis of results corresponded to the index of orientation. The orientation index is a measure of the degree to which the long axis of a cell is aligned with a particular direction or orientation. The orientation index can thus be used to quantify the degree of alignment of cells in our 3D model. To calculate the orientation index, one typically measures the angle between the long axis of a cell and a reference direction. This angle is then divided by 180 degrees to give a value between 0 and 1. A value of 0 indicates that the cell is oriented perpendicular to the reference direction, while a value of 1 indicates that the cell is oriented parallel to the reference direction. The maximum value of the orientation index is 1, and cells were considered aligned if the index was ≥0.5 (value). Therefore, the orientation of the cells, over given distances, could be quantified with high precision.

### 2.7. Beating Characteristics

To investigate the beating characteristics of cardiomyocytes in 3D cultures with and without patterning, neonatal cardiac cells were cultured at a cell density of 10^6^ cells/mL in pre-gel mixtures of dECM-fibrin on patterned and non-patterned substrates. Synchrony and onset of beating was judged visually on a per-well basis.

### 2.8. Statistical Analysis

The data was compared using an unpaired *t*-test (two-tailed, equal variances) and two-way ANOVA test with multiple comparison, and one-way ANOVA test with multiple comparison in the GraphPad software. Error bars represent the mean ± standard deviation (SD) of the measurements (* *p* < 0.05, ** *p* < 0.01, *** *p* < 0.001, and **** *p* < 0.0001).

## 3. Results

### 3.1. 3D and 2D Cultures Show Preferential Alignment and Present Elongated Aspect Regardless of Groove Dimension

Confocal images of the 3D cells cultured inside the hydrogel were acquired and the orientation of the cells in different stacks was observed. Figure 2 pictures the experimental flow aiming to align the cells and the confocal images of the cell alignment in 2D and 3D cell cultures on patterned PDMS chips. Both the 2D and 3D cell cultures, regardless of the groove dimensions, presented an elongated aspect and showed preferential alignment in accordance to the grooves’ direction, as expected [3,33,34,35]. Our analyses show that cells are aligned in the designed pattern in the hydrogel. This orientation propagates to the cells that are not in the direct vicinity with the corners in 2D, and to the bulk of the hydrogel in 3D culture. According to this section, the alignment of the cells in 2D and 3D is qualitatively confirmed, regardless of the groove dimensions. Therefore, the cell alignment is encouraged by the corner of the wall.

### 3.2. The Cell Alignment Propagates in the Grooves Thank to the Contact Guidance

Several stacks of confocal images were used to quantitatively assess cell orientation and propagation away from the microstructured surface. Figure S2 presents the middle stack of the 3D structure and the orientation of the cells, which is in the same orientation in all stacks. In the patterned hydrogel, the cells align even if they are not directly in contact with the aligning microstructures. The alignment propagates into the bulk region of the hydrogel. Cell alignment is also reflected in the shape of nuclei, as the nuclei appear pulled and elongated in the direction of the microgrooves. The relationships between cell alignment and intracellular structure and organization are consistent with previous observations [36].

Secondly, although cellular alignment propagates away from the PDMS microstructures, this propagation effect is limited. We quantified the orientation index (1 = fully aligned, 0 = unordered) in confocal images at different heights from the bottom of the grooves (Figure 3A) and also in single steps (Figure 3D) to determine propagation efficiency. At all heights investigated in the grooves 350 µm × 350 µm, orientation is relatively well maintained (no significant differences in Figure 3B). Using a groove of 350 µm × 350 µm dimensions (Figure 3C), we calculated the distance from the inner corner versus the orientation index. The distance from the inner corner is identical at 50 µm and 300 µm. For better understanding, it is depicted differently in the figure. Hence, the maximum distance from the corner is 391 µm (350 µm from the bottom and 175 µm from the side). The lowest orientation index and the maximum distance from the corner (391 µm) can be found for the middle region of interest (ROI) of the 350 µm stack. The interaction *p*-value in two-way ANOVA analysis is 0.97 which indicates no significant difference between the overall behavior in each group. In light of this, the calculated P-value for row factor and column factor (0.52 and 0.4, respectively) showed no significant difference in orientation index for different distances from the bottom of the groove or the wall corner. Altogether, the measurements demonstrate our capability of aligning cells in 3D structures up to 350 × 350 µm.

We manufactured a groove with a width large enough (2 mm) to be considered as unlimited in one side (Figure 3D) in order to investigate the maximum distance of alignment propagation in 3D hydrogel. Hence, the cells in 3D hydrogels in this structure were just restricted to one side. Figure 3E shows the distance from the inner corner in each confocal stack. The results indicate that an increase in distance from the side wall as well as the bottom of the groove is associated with a gradual decrease of cell orientation. Two-way ANOVA analysis indicated a significant difference between row and column factors (Interaction *p*-value = 0.18, Row factor *p*-value < 0.0001, and Column factor *p*-value < 0.0001). This suggests a strong influence of distance on the alignment structures.

We calculated the distance of each ROI from the inner corner using the vertical and horizontal distance of each ROI to prove the impact of distance from the inner corner on cell alignment at larger distances. This was plotted against the orientation index. Figure 3F illustrates that cell orientation can be maintained up to 380 µm distance from the inner corner of the wall. The alignment of cells significantly decreases at distances larger than 380 µm (*p* = 0.0038).

Contact guidance appears to be sensed from both sides of grooves measuring 350 µm × 350 µm with cells constrained between two close walls. This leads to an extension of the orientation, up to 500 µm distance. As the 350 × 350 µm grooves provide highly aligned 3D cell structures with a diameter comparable to trabeculae carneae, this model was used to further investigate the stability of the molded hydrogel and beating characteristics revealed by the cells in the model.

### 3.3. 3D Patterned Hydrogel Aids a Reliable Measurement of Beating

**Figure 4:**
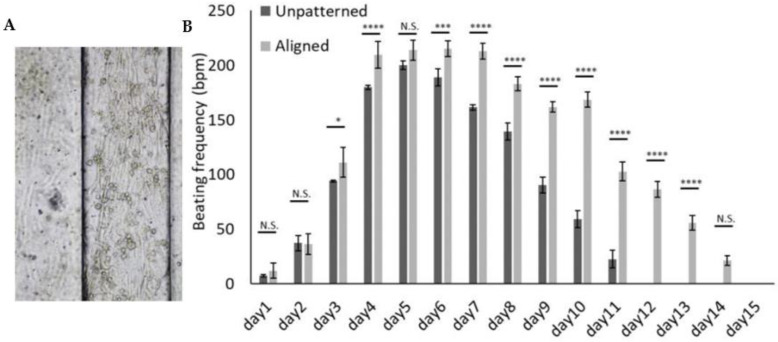
The beating behavior of the cells. (**A**) Neonatal cardiac cell alignment in the microgrooves. (**B**) The comparison between the beating frequency in the 3D dECM-fibrin hydrogel, with and without patterning. Two-way ANOVA with multiple comparison showed significant difference from day 3 between two groups. Scale bar: 100 µm. N.S. = *p* > 0.05, * = *p* ≤ 0.05, *** = *p* ≤ 0.001, **** = *p* ≤ 0.0001.

In order to delineate the beating characteristics in our experimental setting, we compared the beating rate of the neonatal cardiac cells in the patterned hydrogel with the random distribution sample without any pattern. As described in Section 2.7, the beating rate of the cells in both models, with and without patterns, were measured every day for each sample (four replication for each group). Figure 4 displays the alignment of the neonatal cardiac cells in the microgrooves (Figure 4A) and the comparison of beating rate in patterned and non-patterned hydrogel (Figure 4B). The results showed that the frequency of the beating significantly changed from day three between the cells in patterned hydrogel sample and the cells with random distribution in the hydrogel. In both groups, the beating rate was within the range of the physiological beating rate of rat heart. However, the 3D patterned hydrogel was able to maintain beating for a longer period in comparison to the non-patterned sample. The contractility in non-patterned samples lasted for approximately 10 days with the gradual loss of the beating rate from day five. In the patterned sample, the beating faded away through day 14 and it maintained physiological beating until day 10.

### 3.4. Successful Detachability and Stability of the Hydrogel from the PDMS Substrate

**Figure 5:**
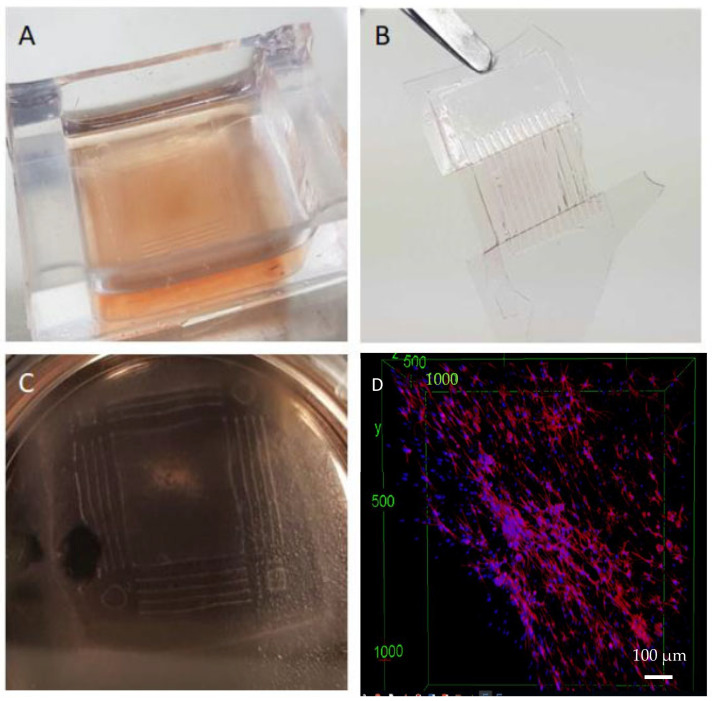
Patterned hydrogel. (**A**) the patterned hydrogel in culture. (**B**) patterned hydrogel once peeled off the PDMS mold. (**C**) Preserved H9c2 cell alignment in the peeled structure. (**D**) the cells are stained with Phalloidin and DAPI to show the alignment stability in the free-standing structure for 7 days after the detachment from the PDMS mold.

Having succeeded in designing the aligned structure, we interrogated the possibility of peeling off the cell-laden hydrogel to reveal a free-standing structure. As shown in Figure 5, the dimensions of this structure can be modified based on the application. To investigate these features, the cardiac cells were cultured in the 3D patterned hydrogel (Figure 5A). Upon removing the hydrogel, we were able to successfully not only handle the sample but also have a stabilized patterned structure (Figure 5B). Furthermore, cells could be successfully cultured in 3D hydrogels, even in the absence of a PDMS support mold for seven days after detaching from the patterned surface (Figure 5C). Last but not least, the confocal imaging of the stained cells visually shows that alignment achieved using microstrutured PDMS grooves can be maintained in the free-standing hydrogel for at least seven days following removal from the PDMS substrate.

## 4. Discussion

In this work, we were able to design and develop an in vitro 3D cardiac model which accurately addresses the dimension and alignment features of native trabeculae carneae structures. We were able to create a functional unit using simple surface hydrogel patterning. The size of the model measured 350 µm × 350 µm × 1 cm in length, which corresponds to the size range of trabeculae carneae. In principle, a groove size of more than 350 µm is less relevant because of the deprivation of nutrients and oxygen for larger structures in the absence of microvasculature [31]. While our study specifically examines the similarities in dimensions and cell alignment to the trabeculae carneae structure in the heart, the model we present can be applied to other cell models as well. The main goal is to showcase the alignment and propagation of cell orientation in a 3D setting.

In this specific study, we chose to use H9C2 cells as an example of a well-established in vitro cardiac model for drug screening, which has the ability to differentiate towards a cardiac phenotype. The reason for using H9C2 cells in this study is to demonstrate the concept of alignment and investigate the maximum distance of alignment, which are the main focus of the paper. H9C2 cells are a useful model, as they are easy to work with and can provide useful insights into the engineering concept. However, when it comes to the functional aspect of the model, we have used neonatal cardiac cells as well, to confirm that the model can improve the functionality of real cardiac cells.

Our examination of cell cytoskeleton morphology by staining the actin filaments of the cardiac cells not only confirmed that cell alignment is not an artifact due to topographical restrictions, but also originates from the cellular response to the guidance. Several studies indicated that cellular orientation leads to long actomyosin bundle formation [37]. F-actin filaments staining of the cardiac cells in the patterned structures (2D and 3D) shows that actomyosin fibers are aligned in the direction of the microfabricated grooves. This actomyosin fiber alignment has also been reported in patterned substrates with subcellular dimensions and in which the spatial constraints are not the problem [38,39,40]. These findings are further supported by nuclear elongation, which was comparable with those reported by other studies showing the cell orientation in a wide range of widths and depths from nano to micro dimensions [32,41,42,43]. We utilized the OrientationJ plugin in the ImageJ software to accurately define the boundaries of the cells and nuclei, which enabled us to quantitatively measure the orientation of the cells. Overall, this confirms a cellular reorganization response due to contact guidance and propagation of the alignment in larger distances. Our data indicates that the grooves’ inner corners are of particular importance in cellular alignment. This provides a hint to the mechanisms involved. Cell orientation was reported to be initiated with cellular sensing of edges of the microgrooves by filopodia [44]. Particularly for cells located in close vicinity to groove surfaces, the contact guidance will cause alignment, as it is known that between surface topography and the remaining general microenvironment, the topographical features have a greater influence on cell alignment [45]. In a 2D setting, the locally aligned cells can serve as a guide for the neighboring cells to induce/promote their alignment [46,47]. Although efficient over many cell diameters, the process of alignment propagation eventually was reported to become inefficient for wider channels (500–800 μm) [46,47]. Our study confirms a 3D orientation process with a similar order of magnitude for the propagation distance, with highly efficient alignment in 350 × 350 μm cross-section channels and progressive loss of orientation for wider grooves. We think that minute cell orientation in the 3D hydrogel can be explained by edge sensing of the cells located near the groove corners, propagating contact guidance to the nearby cells, and, ultimately, the propagation of the alignment in the whole structure.

Cellular contact guidance, however, might not be the only mechanism involved in myocardial cell alignment. Cells in native tissue perform mechanosensing, and cardiomyocytes also perform mechano-electric feedback [48]. This means that cells can sense each other remotely and control their microenvironment, and transfer the deformations through the ECM. Such long-range effects might be particularly important when permitting hydrogel self-condensation as used in the preparation of macroscopic Engineered Heart Tissues (EHT) [26,27]. For local organization/structures up to around 400 μm, contact guidance from suitable surface topography can conveniently be used to induce efficient cell alignment. When addressing size scales beyond, long-range mechanical forces and dynamic ECM reorganization would become more important, imposing the specific constraints on the mechanical work environment experienced by the cells [26,27].

To evaluate the functionality of our 3D in vitro model, we assessed the beating characteristics of cultured neonatal cardiac cells. The neonatal cardiac cells became aligned to the direction of the microgrooves. Their initial beating frequency was not altered and was similar to the one in 3D random cell culture (200 bpm). Importantly, the beating lasted for at least 14 days in aligned structures, which is a significant improvement over the results from the non-patterned samples. These results are in line with several reports showing longer retention of the beating function on patterned as compared with non patterned samples [41,49,50]. Pacemaker channel density in vitro [49] and excitation wave propagation [51] affect the beating characteristics of cardiac cells. It can therefore be concluded that the synchronization and contractility of cardiac cells in vitro are improved by aligning the cells, in addition to the mechanical and physical properties of the hydrogel [29,52]; however, it remains unclear whether this occurs primarily through improved pacemaker channel maintenance or improved propagation of action potentials.

Finally, to investigate the practicality of our model in different applications, such as drug screening, tissue engineering, and heart-on-chip, it is desirable to be able to handle the system without any external support while maintaining cell alignment in the structures. This simplifies the system and makes it independent of the support material. Indeed, the PDMS mold is incompatible with all but very hydrophilic drugs, and it will slowly absorb and release lipophilic small molecules [53]. Further, we expect free-standing structures to facilitate assembly to larger-scale structures, as well as more sophisticated heart-on-chip devices. Altogether, a 3D free-standing structure of the cells is a huge step forward in the construction of 3D heart models.

We would like to clarify that our model is similar to trabeculae carneae structures in terms of cell alignment and dimensions. Our neonatal cardiac cells model is composed of the same cell types as trabeculae carneae structures, as it is isolated from cardiac tissue and comprises a pool of different cell types found in the myocardium. Additionally, our hydrogel is composed of decellularized extracellular matrix and fibrin, which closely mimics the natural environment of cardiac cells in native tissue. Therefore, our model can be considered a relative model to the Trabeculae Carneae. However, it is important to note that to validate the model in more detail, comparing the mechanical and electrical properties of our model and native trabeculae carneae tissue is critical.

## 5. Conclusions

The aim of this paper was to develop a simple method for 3D cell alignment. We showed that cardiac cells self-organized into a 3D aligned structure, without any external force, when encapsulated in micropatterned dECM-fibrin hydrogels. This alignment is due to the contact guidance of the inner corners of the grooves. Seeding cardiomyocytes in grooves of dimensions between 100–350 µm in a 3D cell culture without any other interactions during cell culture was sufficient to obtain highly aligned 3D cardiac cells. Using an infinite groove, we investigated the maximum dimensions of cell alignment propagation, which is up to 250–300 µm from a corner. In addition, the neonatal cardiac cells cultured in this model showed long-term contractility maintenance, indicating that the functionality of the structure had been maintained. The 3D constructs can be detached from the PDMS surfaces, making them a suitable model for drug screening experiments. In this study, we investigated the limitations of current models related to the three-dimensional alignment of cells in hydrogels. Our focus was on utilizing H9c2 cells as a preliminary model to demonstrate the feasibility of the concept. However, we suggest that the model could potentially be applied to other cell types to create aligned tissues, such as muscle, nerve, or tendon tissue. However, further investigation is necessary to fully validate the model by comparing its mechanical and electrical properties with those of the native trabeculae carneae tissue. These limitations and potential avenues for future research should be taken into consideration in future studies in this field. The model can be viewed as a recapitulation of 3D aligned in vitro model, which is a basis for many bioengineering, biology, and pharmaceutical studies, thus making it suitable for a wide range of applications, including drug screening, tissue engineering, and heart-on-chip models.

## Data Availability

Not applicable.

## Supplementary Materials

The following supporting information can be downloaded at: https://www.mdpi.com/article/10.3390/cells12040576/s1, Figure S1: Photograph of a pig heart opened by a frontal incision illustrating trabeculae carneae; Figure S2: The confocal images of the alignment measurement.

## References

[1] Mathur A., Ma Z., Loskill P., Jeeawoody S., Healy K.E. (2016). In vitro cardiac tissue models: Current status and future prospects. Adv. Drug Deliv. Rev..

[2] Kurokawa Y.K., George S.C. (2016). Tissue engineering the cardiac microenvironment: Multicellular microphysiological systems for drug screening. Adv. Drug Deliv. Rev..

[3] Aubin H., Nichol J.W., Hutson C.B., Bae H., Sieminski A.L., Cropek D.M., Akhyari P., Khademhosseini A. (2010). Directed 3D cell alignment and elongation in microengineered hydrogels. Biomaterials.

[4] Helm P., Beg M.F., Miller M.I., Winslow R.L. (2005). Measuring and Mapping Cardiac Fiber and Laminar Architecture Using Diffusion Tensor MR Imaging. Ann. N. Y. Acad. Sci..

[5] Camelliti P., Borg T.K., Kohl P. (2005). Structural and functional characterisation of cardiac fibroblasts. Cardiovasc. Res..

[6] Vasserman I.N., Matveenko V.P., Shardakov I.N., Shestakov A.P. (2015). Numerical simulation of the propagation of electrical excitation in the heart wall taking its fibrous laminar structure into account. Biophysics.

[7] Hsu E.W., Henriquez C.S. (2001). Myocardial fiber orientation mapping using reduced encoding diffusion tensor imaging. J. Cardiovasc. Magn. Reson..

[8] McLean M., Prothero J. (1992). Determination of relative fiber orientation in heart muscle: Methodological problems. Anat. Rec..

[9] Fatemifar F., Feldman M.D., Oglesby M., Han H.-C. (2018). Comparison of Biomechanical Properties and Microstructure of Trabeculae Carneae, Papillary Muscles, and Myocardium in the Human Heart. J. Biomech. Eng..

[10] Halaney D.L., Sanyal A., Nafissi N.A., Escobedo D., Goros M., Michalek J., Acevedo P.J., Pérez W., Escobar G.P., Feldman M.D. (2017). The Effect of Trabeculae Carneae on Left Ventricular Diastolic Compliance: Improvement in Compliance With Trabecular Cutting. J. Biomech. Eng..

[11] Goo S., Joshi P., Sands G., Gerneke D., Taberner A., Dollie Q., LeGrice I., Loiselle D. (2009). Trabeculae carneae as models of the ventricular walls: Implications for the delivery of oxygen. J. Gen. Physiol..

[12] Han J.-C., Taberner A.J., Nielsen P.M.F., Loiselle D.S. (2013). Interventricular comparison of the energetics of contraction of trabeculae carneae isolated from the rat heart. J. Physiol..

[13] Sands G., Goo S., Gerneke D., LeGrice I., Loiselle D. (2011). The collagenous microstructure of cardiac ventricular trabeculae carneae. J. Struct. Biol..

[14] Kolewe M.E., Park H., Gray C., Ye X., Langer R., Freed L.E. (2013). 3D Structural Patterns in Scalable, Elastomeric Scaffolds Guide Engineered Tissue Architecture. Adv. Mater..

[15] Eyckmans J., Boudou T., Yu X., Chen C.S. (2011). A Hitchhiker’s Guide to Mechanobiology. Dev. Cell.

[16] Nikkhah M., Edalat F., Manoucheri S., Khademhosseini A. (2012). Engineering microscale topographies to control the cell–substrate interface. Biomaterials.

[17] Stevens M.M., Stevens M.M., George J.H. (2005). Exploring and Engineering the Cell Surface Interface. Science.

[18] Grosberg A., Alford P.W., McCain M.L., Parker K.K. (2011). Ensembles of engineered cardiac tissues for physiological and pharmacological study: Heart on a chip. Lab Chip.

[19] Bursac N., Parker K., Iravanian S., Tung L. (2002). Cardiomyocyte Cultures With Controlled Macroscopic Anisotropy. Circ. Res..

[20] Au H.T.H., Cui B., Chu Z.E., Veres T., Radisic M. (2009). Cell culture chips for simultaneous application of topographical and electrical cues enhance phenotype of cardiomyocytes. Lab Chip.

[21] Annabi N., Selimovic S., Cox J.P.A., Ribas J., Bakooshli M.A., Heintze D., Weiss A., Cropek D., Khademhosseini A. (2013). Hydrogel-coated microfluidic channels for cardiomyocyte culture. Lab Chip.

[22] Mosiewicz K.A., Kolb L., Van Der Vlies A.J., Martino M., Lienemann P., Hubbell J., Ehrbar M., Lutolf M.P. (2013). In situ cell manipulation through enzymatic hydrogel photopatterning. Nat. Mater..

[23] Norman J.J., Desai T.A. (2005). Control of Cellular Organization in Three Dimensions Using a Microfabricated Polydimethylsiloxane–Collagen Composite Tissue Scaffold. Tissue Eng..

[24] Yang S., Min J.H., Cho K., Seo I.H., Ryu W., Koh W.-G. (2019). Fabrication of microgrooved scaffolds using near-field electrospinning-assisted lithography (NFEAL). J. Ind. Eng. Chem..

[25] Engelmayr G.C., Cheng M., Bettinger C.J., Borenstein J.T., Langer R., Freed L.E. (2008). Accordion-like honeycombs for tissue engineering of cardiac anisotropy. Nat. Mater..

[26] Zimmermann W.-H., Schneiderbanger K., Schubert P., Didié M., Münzel F., Heubach J., Kostin S., Neuhuber W., Eschenhagen T. (2002). Tissue Engineering of a Differentiated Cardiac Muscle Construct. Circ. Res..

[27] Zimmermann W.-H., Melnychenko I., Wasmeier G.H., Didié M., Naito H., Nixdorff U., Hess A., Budinsky L., Brune K., Michaelis B. (2006). Engineered heart tissue grafts improve systolic and diastolic function in infarcted rat hearts. Nat. Med..

[28] Li Y., Huang G., Zhang X., Wang L., Du Y., Lu T.J., Xu F. (2014). Engineering cell alignment in vitro. Biotechnol. Adv..

[29] Navaee F., Renaud P., Kleger A., Braschler T. (2023). Highly Efficient Cardiac Differentiation and Maintenance by Thrombin-Coagulated Fibrin Hydrogels Enriched with Decellularized Porcine Heart Extracellular Matrix. Int. J. Mol. Sci..

[30] Fatemi Far S., Feldman M., Han H.-C. Characterization of Biomechanical Properties of Human Trabeculae Carneae. Proceedings of the Bioengineering and Biotransport Conference.

[31] de Mello R.A., Mountzios G., Tavares Á.A. (2015). International Manual of Oncology Practice: (iMOP)-Principles of Medical Oncology.

[32] Smith A.S.T., Yoo H., Yi H., Ahn E.H., Lee J.H., Shao G., Nagornyak E., Laflamme M.A., Murry C.E., Kim D.-H. (2017). Micro- and nano-patterned conductive graphene–PEG hybrid scaffolds for cardiac tissue engineering. Chem. Commun..

[33] Uttayarat P., Chen M., Li M., Allen F.D., Composto R.J., Lelkes P.I. (2008). Microtopography and flow modulate the direction of endothelial cell migration. Am. J. Physiol. Circ. Physiol..

[34] Cortella L.R.X., Cestari I.A., Lahuerta R.D., Araña M.C., Soldera M., Rank A., Lasagni A.F., Cestari I.N. (2021). Conditioning of hiPSC-derived cardiomyocytes using surface topography obtained with high throughput technology. Biomed. Mater..

[35] Litowczenko J., Maciejewska B.M., Wychowaniec J.K., Kościński M., Jurga S., Warowicka A. (2019). Groove-patterned surfaces induce morphological changes in cells of neuronal origin. J. Biomed. Mater. Res. Part A.

[36] Hwang Y., Seo T., Hariri S., Choi C., Varghese S. (2017). Matrix Topographical Cue-Mediated Myogenic Differentiation of Human Embryonic Stem Cell Derivatives. Polymers.

[37] Prager-Khoutorsky M., Lichtenstein A., Krishnan R., Rajendran K., Mayo A., Kam Z., Geiger B., Bershadsky A.D. (2011). Fibroblast polarization is a matrix-rigidity-dependent process controlled by focal adhesion mechanosensing. Nat. Cell Biol..

[38] Cabezas M.D., Meckes B., Mirkin C.A., Mrksich M. (2019). Subcellular Control over Focal Adhesion Anisotropy, Independent of Cell Morphology, Dictates Stem Cell Fate. ACS Nano.

[39] Sniadecki N.J., Desai R.A., Ruiz S.A., Chen C. (2006). Nanotechnology for Cell–Substrate Interactions. Ann. Biomed. Eng..

[40] Baptista D., Teixeira L., van Blitterswijk C., Giselbrecht S., Truckenmüller R. (2019). Overlooked? Underestimated? Effects of Substrate Curvature on Cell Behavior. Trends Biotechnol..

[41] Tijore A., Irvine S.A., Sarig U., Mhaisalkar P., Baisane V., Venkatraman S.S. (2017). Contact guidance for cardiac tissue engineering using 3D bioprinted gelatin patterned hydrogel. Biofabrication.

[42] Naseer S.M., Manbachi A., Samandari M., Walch P., Gao Y., Zhang Y.S., Davoudi F., Wang W., Abrinia K., Cooper J.M. (2017). Surface acoustic waves induced micropatterning of cells in gelatin methacryloyl (GelMA) hydrogels. Biofabrication.

[43] Kankala R.K., Zhu K., Sun X.-N., Liu C.-G., Wang S.-B., Chen A.-Z. (2018). Cardiac Tissue Engineering on the Nanoscale. ACS Biomater. Sci. Eng..

[44] You R., Li X., Luo Z., Qu J., Li M. (2015). Directional cell elongation through filopodia-steered lamellipodial extension on patterned silk fibroin films. Biointerphases.

[45] Yim E.K., Darling E.M., Kulangara K., Guilak F., Leong K.W. (2010). Nanotopography-induced changes in focal adhesions, cytoskeletal organization, and mechanical properties of human mesenchymal stem cells. Biomaterials.

[46] Chou C.-L., Rivera A.L., Williams V., Welter J.F., Mansour J.M., Drazba J.A., Sakai T., Baskaran H. (2017). Micrometer scale guidance of mesenchymal stem cells to form structurally oriented large-scale tissue engineered cartilage. Acta Biomater..

[47] Salick M.R., Napiwocki B.N., Sha J., Knight G.T., Chindhy S.A., Kamp T.J., Ashton R.S., Crone W.C. (2014). Micropattern width dependent sarcomere development in human ESC-derived cardiomyocytes. Biomaterials.

[48] Izu L.T., Kohl P., Boyden P.A., Miura M., Banyasz T., Chiamvimonvat N., Trayanova N., Bers D.M., Chen-Izu Y. (2020). Mechano-electric and mechano-chemo-transduction in cardiomyocytes. J. Physiol..

[49] Sartiani L., Bettiol E., Stillitano F., Mugelli A., Cerbai E., Jaconi M.E. (2007). Developmental Changes in Cardiomyocytes Differentiated from Human Embryonic Stem Cells: A Molecular and Electrophysiological Approach. Stem Cells.

[50] van Spreeuwel A.C.C., Bax N.A.M., Bastiaens A.J., Foolen J., Loerakker S., Borochin M., van der Schaft D.W.J., Chen C.S., Baaijens F.P.T., Bouten C.V.C. (2014). The influence of matrix (an)isotropy on cardiomyocyte contraction in engineered cardiac microtissues. Integr. Biol..

[51] Ponard J.G., Kondratyev A.A., Kucera J.P. (2007). Mechanisms of Intrinsic Beating Variability in Cardiac Cell Cultures and Model Pacemaker Networks. Biophys. J..

[52] Ahearne M. (2014). Introduction to cell–hydrogel mechanosensing. Interface Focus.

[53] van Meer B., de Vries H., Firth K., van Weerd J., Tertoolen L., Karperien H., Jonkheijm P., Denning C., Ijzerman A., Mummery C. (2017). Small molecule absorption by PDMS in the context of drug response bioassays. Biochem. Biophys. Res. Commun..

