# Supplementary material for: A Three-Dimensional Engineered Cardiac In Vitro Model: Controlled Alignment of Cardiomyocytes in 3D Microphysiological Systems
Source: Cells. 2023 Feb 10;12(4):576. doi: 10.3390/cells12040576 (PMC9954012; doi:10.3390/cells12040576)
Supplement: Supplementary file 1 [file cells-12-00576-s001.zip › Supplementary figure S1.pdf]

Supplementary Figure S1. Photograph of a pig heart opened by a frontal incision illustrating trabeculae carneae.

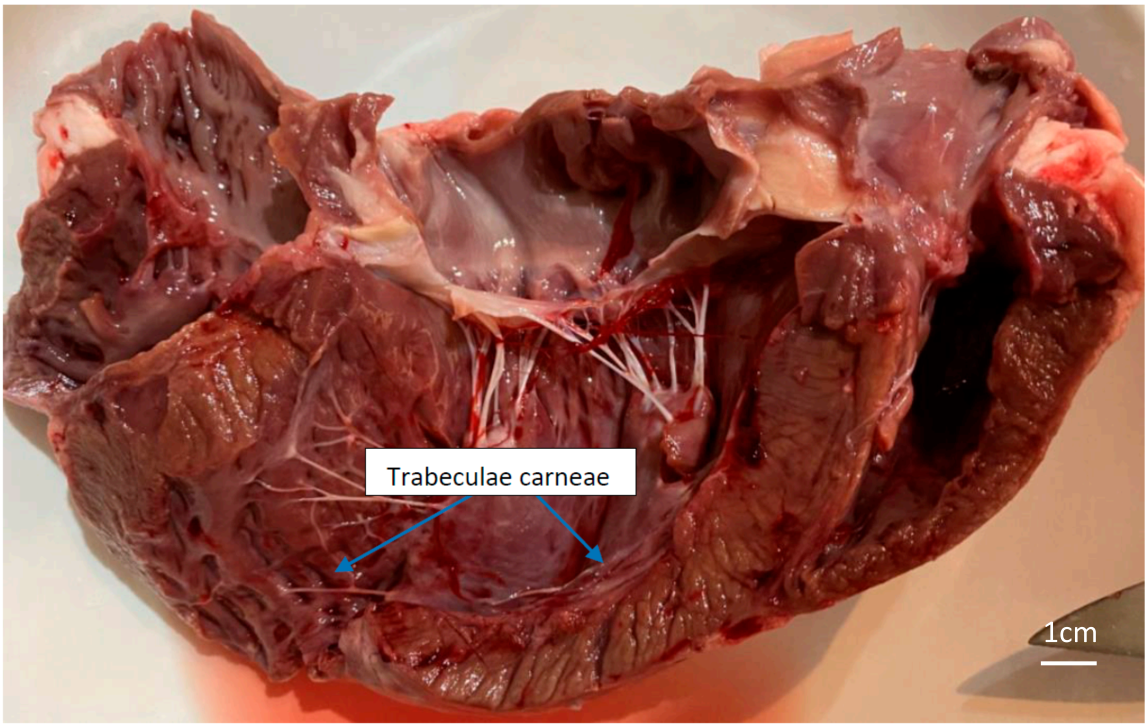

Figure S1. Photograph of a opened by a frontal incision illustrating the trabeculae carneae.
