# Supplementary material for: A Three-Dimensional Engineered Cardiac In Vitro Model: Controlled Alignment of Cardiomyocytes in 3D Microphysiological Systems
Source: Cells. 2023 Feb 10;12(4):576. doi: 10.3390/cells12040576 (PMC9954012; doi:10.3390/cells12040576)
Supplement: Supplementary file 1 [file cells-12-00576-s001.zip › Supplementary figure S2.pdf]

Supplementary Figure S2:

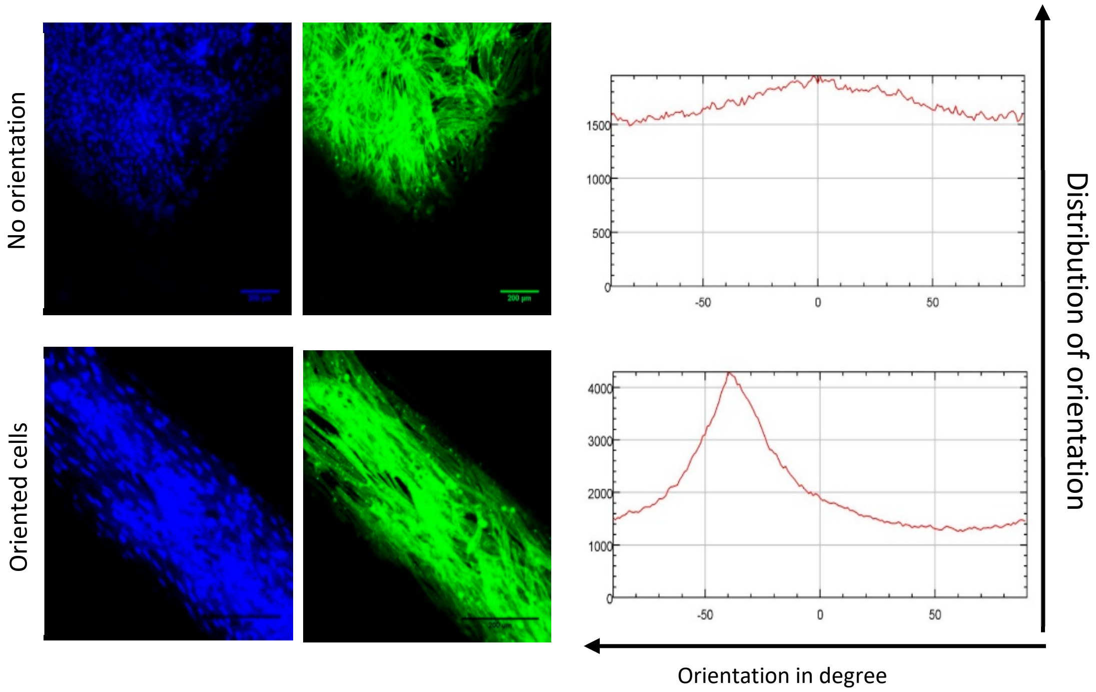

The confocal images of the alignment measurement of H9c2in co-culture with fibroblasts in A) 3D culture in hydrogel, on flat substrate (no grooves), and B) 3D culture in hydrogel, inside a 350 μm wide groove (at h=175 μm). Phalloidin staining in green shows the alignment and organization of the actin filaments in the cells. Nuclei staining with DAPI in blue displays the elongation and orientation of nuclei in patterned substrate. The middle stack of the confocal images (h=175 μm) has been selected for this alignment observation. Scale bar: 200 μm.
